# Oral dysbiosis in children with renal failure: a systematic review

**DOI:** 10.3389/fdmed.2026.1832651

**Published:** 2026-08-27

**Authors:** Juan Antonio Ruiz-Roca, María Pilar Pecci-Lloret, Antonio Romano, Francesco Carinci, Dorina Lauritano, Nuria Pérez-Guzmán, Alberta Lucchese

**Affiliations:** 1 Dermatology, Stomatology, Radiology and Physical Medicine, Hospital Morales Meseguer, Medicine School, University of Murcia, Murcia, Spain; 2 Multidisciplinary Department of Medical-Surgical and Dental Specialties, University of Campania “Luigi Vanvitelli”, Naples, Italy; 3 Department of Translational Medicine, University of Ferrara, Ferrara, Italy

**Keywords:** children, chronic kidney disease, oral dysbiosis, oral microbiome, renal failure

## Abstract

**Introduction:**

The oral microbiome plays an important role in maintaining host homeostasis and may interact with systemic diseases through the oral–systemic axis. Chronic kidney disease (CKD) in childhood is associated with chronic inflammation, metabolic alterations, and immune dysfunction, which may influence microbial ecosystems. While intestinal dysbiosis has been widely studied in paediatric CKD, alterations in the oral microbiome remain less well understood. The aim of this study was to qualitatively synthesize evidence on oral microbiome alterations in children and adolescents with CKD compared with systemically healthy controls.

**Methods:**

A systematic review was conducted according to PRISMA 2020 and registered in PROSPERO (CRD420261334000). PubMed, Scopus, Web of Science, Cochrane, and SciELO were searched (February 24, 2026). Eligible studies included participants aged 0–18 years with CKD (any stage), dialysis, or kidney transplantation and a healthy control group. Risk of bias was assessed using JBI and ROBINS-I v2 tools.

**Results:**

From 109 records, 7 studies were included. Most used saliva, tongue/oral swabs, or dental plaque, and 16S rRNA sequencing was the most frequent method. Five studies reported oral dysbiosis in CKD, including severe dysbiosis grades in adolescents with end-stage disease, while two studies reported no significant differences versus controls.

**Conclusion:**

Findings on individual taxa were inconsistent across studies, likely due to heterogeneity in renal phenotype, oral niche sampled, and analytical methods. Evidence suggests that salivary biochemical alterations (notably urea and pH) and inflammatory burden may influence microbial composition, whereas the tongue microbiome may show relative ecological stability in some paediatric cohorts. Children and adolescents with CKD may present oral microbiome alterations, but current evidence is limited by methodological heterogeneity and moderate-to-high risk of bias. Standardized sampling and longitudinal multicentre studies are needed to determine robust microbial signatures and their value as non-invasive biomarkers in paediatric nephrology.

**Systematic Review Registration:**

https://www.crd.york.ac.uk/PROSPERO/view/CRD420261334000.

## 1 Introduction

The human body harbours between 10 and 100 trillion microorganisms, including bacteria, viruses, fungi and archaea, which constitute complex ecosystems with essential functions for host homeostasis (1). The oral cavity represents one of the most diverse microbial niches in the human body and constitutes a heterogeneous ecosystem known as the oral microbiome, with more than 700 bacterial species described in the oral cavity. This makes it the second most prevalent microbial ecosystem after the gut microbiome (2), playing an important role in maintaining health (3).

Chronic Kidney disease (CKD), characterized by chronic inflammation, oxidative stress, metabolic alterations and immune dysfunction, is associated with high morbidity and mortality, especially in advanced stages and in patients requiring dialysis (4, 5). According to data from the International Society of Nephrology (ISN), According to data from the International Society of Nephrology (ISN), approximately 850 million adults worldwide are affected by CKD, with a proportion of these patients progressing to end-stage renal disease and requiring renal replacement therapy, including dialysis or kidney transplantation, to sustain life (6). In Europe, the annual incidence of stage 3–5 CKD in children and adolescents is estimated at 11–12 cases per million, with an approximate prevalence of 60–70 cases per million in the paediatric population (10). The most common causes of CKD in children include congenital abnormalities of the kidney and urinary tract and hereditary diseases (4).

Many studies suggest that the composition of the salivary microbiome may interact with gut microbial function, influencing host immune responses, regulating the balance between pro-inflammatory and anti-inflammatory processes, and inhibiting the adhesion and invasion of pathogens and metabolic status (7).

When this ecological balance is disrupted, it favours the expansion of opportunistic microorganisms, generating a state of dysbiosis (2).

Oral dysbiosis has been linked not only to oral diseases, but also to systemic diseases such as cardiovascular disease, diabetes and neurodegenerative disorders. In this context, some recent studies have described associations between oral dysbiosis and Alzheimer's disease (2), Parkinson's disease (8) and depression (9), consolidating the concept of the oral-systemic axis.

In some glomerular kidney diseases such as IgA nephropathy, multiniche dysbiosis (oral, pharyngeal, intestinal, and urinary) has been described, with an increase in opportunistic pathogens in the oral cavity and pharynx and a positive association with serum creatinine and urea (10), suggesting a possible interaction between the mucosal microbiome and renal damage or failure.

In the paediatric population, the development of the microbiome has dynamic and environment-dependent characteristics. Although intestinal dysbiosis has been described in children with advanced CKD and renal therapy (11, 12), considerably less is known about the oral microbiome. This distinction is clinically relevant because the oral cavity contains multiple specialised ecological niches that are directly influenced by salivary composition, oral hygiene, dental disease and systemic metabolic alterations.

The initial establishment of the microbiome is influenced by the mode of delivery, breastfeeding, and environmental factors (12). Compared to adults, children have lower gut microbial diversity and functional differences associated with metabolic and growth processes (12).

In relation to the oral cavity, colonisation begins at birth and evolves into a more complex structure with tooth eruption and the formation of differentiated ecological niches (4).

In paediatric patients with end-stage renal disease, significant alterations in the gut microbiome have been reported, including a decrease in Firmicutes and Actinobacteria in peritoneal dialysis and an increase in Bacteroidetes in haemodialysis (11).

Overall, kidney disease in childhood develops in a context of a maturing immune system, an evolving microbiome, and prolonged exposure to drug treatments, restrictive diets, and, in some cases, renal replacement therapies (11, 13). While intestinal dysbiosis in paediatric CKD is well documented (11, 14), the available evidence suggests that certain oral niches, such as the tongue, may maintain remarkable ecological resilience (4, 15). It has also been found that the microbiota in healthy patients is very different from that in patients with actual disease or on dialysis (16).

The aim of this systematic review was to qualitatively synthesize the available evidence on alterations in the oral microbiome of children and adolescents with chronic kidney disease compared with systemically healthy controls.

## 2 Materials and methods

This systematic review was conducted in accordance with the PRISMA 2020 guidelines (17). It has been registered in the PROSPERO (CRD420261334000).

### 2.1 Eligibility criteria

The inclusion criteria were: observational studies (cross-sectional, case control cohort), non-randomized interventional studies evaluating microbiome changes and human studies, participants aged ≤18 years; a clinical diagnosis of chronic kidney disease (CKD) at any stage, undergoing dialysis, or kidney transplantation; inclusion of a comparison group consisting of systemically healthy controls without renal disease; assessment of the oral microbiome; publication in English or Spanish; and no restriction on year of publication.

The exclusion criteria were: studies including adult populations (>18 years) without separate pediatric data; studies exclusively evaluating gut or non-oral microbiota; case reports, case series with fewer than 10 participants, reviews, animal studies or thesis; studies in which chronic kidney disease was not clearly defined or clinically diagnosed; studies including participants with systemic conditions known to significantly affect the oral microbiome (uncontrolled diabetes, immunodeficiency unrelated to CKD), studies lacking a healthy control group.

The inclusion criteria followed the PECO (Population; Exposure; Comparison; Outcome) model:

• P (Population): Children and adolescents (0–18 years)
• E (Exposure): Diagnosis of chronic kidney disease (any stage), dialysis, or kidney transplantation.
• C (Comparison): Systemically healthy children/adolescents without renal disease.
• O (Outcomes): Alterations in the oral microbiome: microbial diversity (*α*- and *β*-diversity), dysbiosis grading, relative abundance of oral taxa, presence of periodontopathogenic bacteria or oral inflammatory biomarkers associated with microbial alterations.

Research question: Do children and adolescents with chronic kidney disease present alterations in the oral microbiome compared with healthy patients?

### 2.2 Information sources and search strategy

#### 2.2.1 Information sources

An electronic search was conducted on 24th February 2026, across different databases: PubMed (Medline), SciELO, Scopus, Cochrane and Web of Science. The process of searching, selecting, data extraction, and quality analysis was carried out by MPPL and NPG, in case of discrepancies, a third reviewer was asked (JARR).

#### 2.2.2 Search terms

The search was performed using the following keywords:

The search formula used across Pubmed (Medline) were:

(“chronic kidney disease” OR CKD OR “renal disease” OR “renal failure” OR nephritis OR dialysis) AND (“oral microbiome” OR “oral microbiota” OR “oral microflora” OR “salivary microbiota” OR “salivary microbiome” OR “tongue microbiota” OR “dental plaque” OR “supragingival plaque”) AND (child* OR pediatric* OR paediatric* OR adolescent* OR “school-age*” OR infant*) NOT (gut OR intestinal)

The common search formula used across SciELO, Scopus, Cochrane and Web of Science were:

(“chronic kidney disease” OR CKD OR “renal disease” OR “renal failure” OR nephritis OR dialysis) AND (“oral microbiome” OR “oral microbiota” OR “oral microflora” OR “salivary microbiota” OR “salivary microbiome” OR “tongue microbiota” OR “dental plaque” OR “supragingival plaque”) AND (child OR pediatric OR paediatric OR adolescent OR “school-age” OR infant) NOT (gut OR intestinal).

### 2.3 Study selection

The search results were exported to EndNote by Clarivate Analytics. From the total number of articles, duplicates were automatically removed using the reference manager, and any remaining duplicates not detected by the software were subsequently discarded manually.

Next, the titles and abstracts of the remaining articles were screened. If they did not meet the previously established inclusion criteria, they were excluded. The remaining articles were then assessed through full-text reading.

### 2.4 Quality Assessment

Analytical cross-sectional studies were evaluated using the Joanna Briggs Institute (JBI) Critical Appraisal Checklist for Analytical Cross-Sectional Studies (18).

Non-randomized interventional studies were assessed using the Risk Of Bias In Non-randomized Studies of Interventions (ROBINS-I v2) tool, updated in November 2025 (19). Based on the respective tools, studies were classified as low, moderate, or high (JBI), and as low, moderate, serious, or critical risk of bias (ROBINS-I v2).

Studies classified as high risk of bias were excluded from the primary data synthesis in order to reduce the influence of substantial methodological limitations on the overall conclusions.

### 2.5 Data extraction

The following variables were collected:

(1) Study characteristics (author, year, country, study design).
(2) Participant characteristics (sample size, age, renal disease type, dialysis or transplantation status).
(3) Type of oral sample analyzed (saliva, plaque, tongue coating, gingival crevicular fluid).
(4) Microbiological assessment method (16S rRNA sequencing, PCR, culture-based methods, chromatography–mass spectrometry).
(5) Reported microbial taxa and diversity indices (alpha and beta diversity when available).
(6) Reported statistically significant differences in microbial composition, diversity indices, or relative abundance between CKD patients and healthy controls.

## 3 Results

### 3.1 Study selection and PRISMA flow diagram

**Figure 1:**
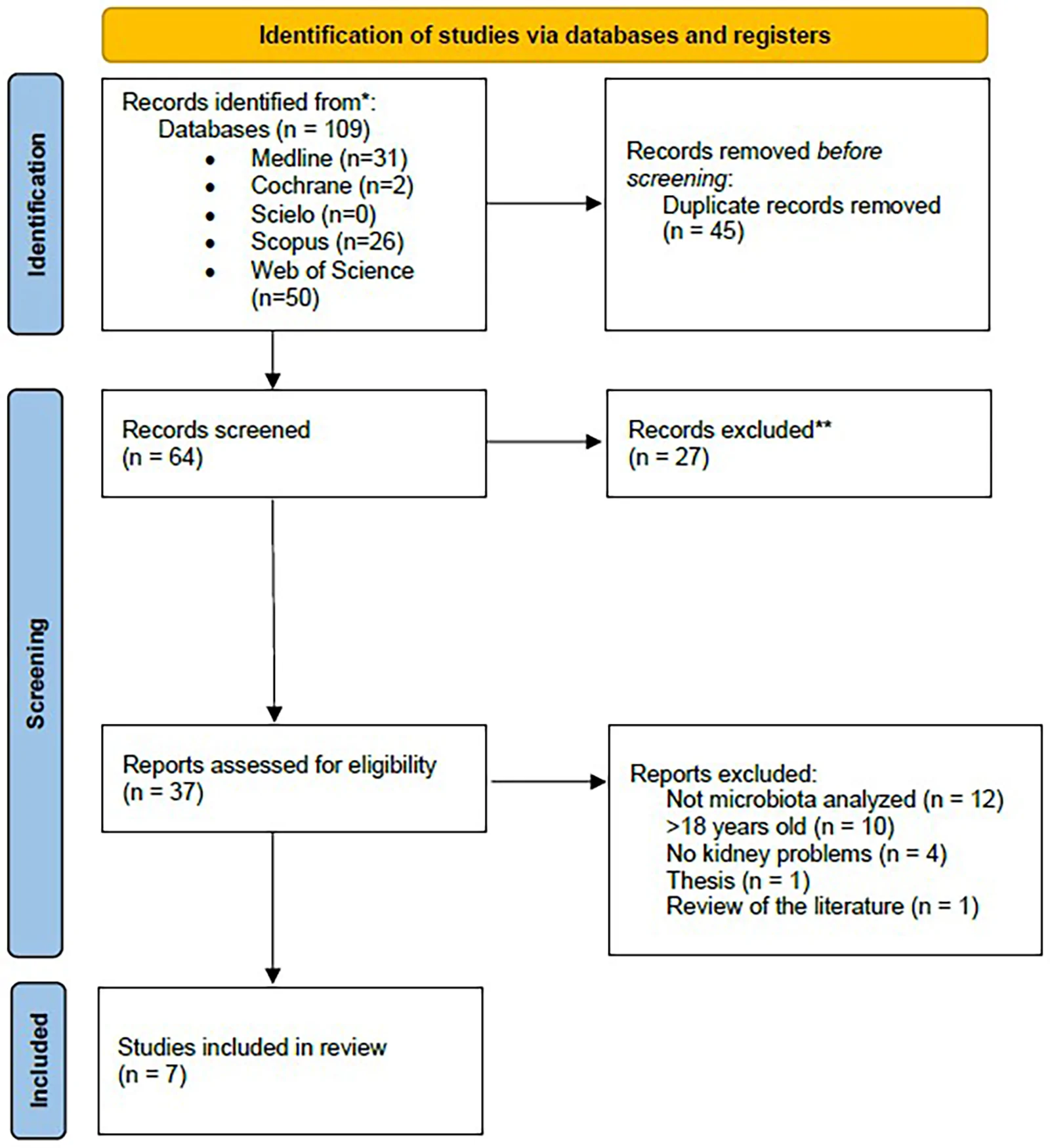
Flow diagram PRISMA 2020.

After obtaining 109 articles from 5 databases, the duplicated were removed before screening (45), leaving a total of 64 records screened. Evaluating title and abstract, 27 records were excluded and 37 reports were assessed for eligibility. From the 37 reports, 30 were excluded, in 12 microbiota were not analyzed, in 10, they were patients more than 18 years old, 4 did not have kidney problems, 1 was a thesis and 1 was a review of the literature. Finally, 7 articles were included in this review (Figure 1).

### 3.2 Risk of bias assessment

The JBI used to determine the risk of bias of the cross-sectional studies (Table 1). Of the six selected articled, one showed a high risk of bias (20), four showed a moderate risk of bias (4, 21–23), and one a low moderate risk of bias (24).

**Table 1:** JBI checklist results.

Article	1	2	3	4	5	6	7	8	Overall
Hoefer et al. (4)	Y	Y	Y	Y	P	N	Y	Y	Moderate
Cesar Alves et al. (20)	Y	P	Y	P	N	N	P	N	High
Al-Nowaiser et al. (22)	Y	Y	Y	Y	P	P	Y	Y	Moderate
Chen et al. (24)	Y	Y	Y	Y	Y	Y	Y	Y	Low
Pang et al. (21)	Y	P	Y	Y	P	P	Y	P	Moderate
Morozova et al. (23)	Y	Y	Y	P	P	N	Y	Y	Moderate

Y, Yes; P, Partial, N: No.

The risk of bias of one non-randomized interventional studies was assessed using the Risk Of Bias In Non-randomized Studies of Interventions (ROBINS-I v2) (Table 2) and this articled showed a high risk of bias (25).

**Table 2:** Robins-I v2 checklist results.

Article	1	2	3	4	5	6	7	Overall
Yang et al. (25)	H	H	L	M	M	L	M	High

H, High; M, Moderate; L, Low.

### 3.3 Characteristics and findings of the included studies

Table 3 presents the demographic and clinical characteristics of the participants, including sample size, age, renal diagnosis and dialysis or transplantation status.

**Table 3:** Articles data extracted.

Article	Sample size	Age	Renal disease type	Dialisys or transplantation status
Pang et al. (21)	34 children in total (20 with HSPN and 14 healthy controls)	HSPN group = 10.56 ± 1.73 years; healthy control grou*p* = 11.87 ± 1.66 years	Henoch-Schönlein purpura nephritis (HSPN)	The patients were not on dialysis or had received a transplant
Chen et al. (24)	164 children in total (98 patients with HSP and 66 healthy controls)	Children under 18 years of age. The HSP group was matched to the control group in terms of age.	Henoch-Schönlein purpura (HSP). Patients were classified as “mixed type” if they presented with more than two clinical manifestations, including renal involvement (hematuria/proteinuria).	Not mentioned
Sergeevna Morozova et al. (23)	50 children and teenagers	Between 12 and 17 years old. Study grou*p* = 13.4 ± 1.8 years; control grou*p* = 13.7 ± 1.9 years	End-stage renal disease (ESKD)	Group 1 included patients with stage 5 ESKD and kidney transplant patients receiving immunosuppressive therapy
Al-Nowaiser et al. (22)	A total of 140 children were included [70 with chronic kidney disease (CKD) and 70 healthy controls]. A subsample of 25 children with CKD and 25 controls was used for microbiological analysis.	study group: 11.4 years (SD 3.4), range 4–13.6 years; control group: 11.5 years (SD 3.4)	Chronic kidney disease (CKD), with primary diagnoses such as pyelonephritis, congenital hypoplasia, hereditary nephropathy, and multisystem diseases	28 children were receiving peritoneal dialysis, 16 hemodialysis, and 26 were not on dialysis.
Cesar Alves et al. (20)	30 subjects	Teenagers and young adults between 12 and 18 years old	Chronic kidney disease (CKD) in stages 1–5.	Not specified
Hoefer et al. (4)	30 young patients with CKD and 21 healthy mothers as a control group	Patients: 14.2 years on average (range 6–25 years); Mothers: 40.8 years on average.	CKD stages 1–5 (CAKUT, glomerulopathy, ciliopathy, systemic disease and others)	7 patients under conservative treatment, 2 on dialysis and 21 post-transplant
Yang et al. (25)	45 pediatric patients	From 3 to 13 years old	Henoch-Schönlein Purple (HSP)	Not mentioned

As shown in Table 4, the oral niches examined also varied considerably. Oral or lingual swabs were the most frequently used diagnostic methods, reported in four studies (4, 21, 22, 24), followed by saliva (20, 22, 23) and dental plaque (20, 22, 23, 25). Only one study analyzed oral mucosa and gingival crevicular fluid (23).

**Table 4:** Articles data extracted 2.

Article	Type of oral sample analyzed	Microbiological assesment method	Reported microbial taxa and diversity indices	Reported microbial taxa and diversity differences
Pang et al. (21)	Thick white coating samples in patients with HSPN and thin white coating in healthy controls	Sequencing of the 16S rRNA gene (hypervariable regions V3–V4) using the Illumina MiSeq platform The process included PCR amplification of these regions prior to sequencing.	-Alpha diversity indices: The ACE, Chao, Shannon, and Simpson indices were reported. -Beta diversity: This was assessed using principal component analysis (PCA), non-metric multidimensional scaling (NMDS), and partial least squares discriminant analysis (PLS-DA). -Major taxa: The dominant orders (over 80%) were Bacteroidales, Selenomonadales, Lactobacillales, Fusobacteriales, Neisseriales, and Actinomycetales. -At the genus level, the most frequent included Prevotella_7, Veillonella, Streptococcus, Neisseria, Leptotrichia, Actinomyces, Fusobacterium, Haemophilus, Prevotella, Rothia and Porphyromonas	-Diversity and indices: Microbial richness (ACE and Chao indices) was significantly lower in the HSPN group (*P* = 0.001 and *P* = 0.004). Diversity (Shannon and Simpson indices) was also drastically reduced in children with HSPN compared to controls (*P* = 0.001 and *P* = 0.005). -Microbial composition: PCA, NMDS, and PLS-DA analyses showed a marked separation in the bacterial community structure between the two groups. -Relative abundance (Taxa): At the order level, the proportions of Clostridiales and Campylobacterales were significantly lower in the HSPN group. At the genus level, Prevotella was less abundant in the HSPN group, while Eikenella showed a significantly higher abundance in children with the disease (*P* < 0.05). LEfSe analysis identified multiple reduced biomarkers in HSPN, such as the Lachnospiraceae and Campylobacteraceae families.
Chen et al. (24)	Oral swab. Samples were obtained by rubbing the inside of both cheeks with an oral swab.	Sequencing of the 16S rRNA gene (hypervariable region V1–V2) using the Illumina HiSeq 2500 platform	-Alpha diversity: Observed OTU indices (richness) and the Shannon index (diversity) were used. -Beta diversity: Assessed using principal coordinates analysis (PCoA) based on weighted and unweighted UniFrac distances. -Dominant taxa: At the phylum level: Firmicutes, Proteobacteria, and Bacteroidetes (representing more than 90% of the total). At the genus level: Streptococcus and Acinetobacter were the most prominent in all individuals.	-Microbial diversity: Children with HSP showed significantly greater richness (observed OTU, *P* < 0.01) and diversity (Shannon, *P* < 0.0001) than healthy controls. -Community structure: PCoA analysis and the ANOSIM test confirmed that the oral microbiota structure in children with HSP differed significantly from that of healthy children (*P* = 0.0001). -Taxon abundance (LEfSe): Enriched in HSP: Phylum Bacteroidetes and genera Neisseria, Veillonella, and Prevotella. Decreased in HSP (higher in healthy individuals): Phylum Proteobacteria and genera Acinetobacter and Haemophilus. -Clinical correlations: The abundance of Prevotella nanceiensis was positively correlated with IgA levels. Prevotella showed a positive correlation with IgM. Haemophilus showed a negative correlation with IgE and IgM, but a positive correlation with the length of hospital stay.
Sergeevna Morozova et al. (23)	Saliva, gingival crevicular fluid (GCF), oral mucosa, and dental plaque.	-Gas chromatography-mass spectrometry (GC-MS): Used for the molecular identification of microorganisms and molecular markers in the analyzed sample (G.A. Osipov method). -Polymerase chain reaction (PCR): Specifically used for the detection of periodontopathogenic bacteria.	-Taxa identified in patients with ESKD: Contamination by opportunistic species such as Bifidobacterium spp., Clostridium difficile, Nocardia asteroides, Rhodococcus spp., Staphylococcus aureus, Alcaligenes spp., Eggerthella lenta, and Candida spp. was found. -Transient microorganisms: Pseudomonas aeruginosa, Klebsiella pneumoniae, Haemophilus parainfluenzae, Gemella morbillorum, Abiotrophy defectia, and Herpes simplex. -Periodontopathogenic bacteria: Tannerella forsythia (red complex), Aggregatibacter actinomycetemcomitans (green complex), and Fusobacterium nucleatum (orange complex). Diversity indices: The study does not explicitly mention “alpha” or “beta” diversity indices, but uses a five-grade scale to assess the severity of oral dysbiosis (Grade 1: normal; Grade 5: severe dysbiosis with yeast-like fungi).	-Microbial composition: The ESKD group showed a significantly higher presence of opportunistic taxa such as Bifidobacterium spp., C. difficile, Nocardia asteroides, Rhodococcus spp., S. aureus, Alcaligenes spp., and Candida spp. (*p* < 0.05) compared to the control group. -Severity of dysbiosis: Significant differences were found in the degrees of dysbiosis; 46.7% of adolescents with ESKD presented with Grade 4 dysbiosis and 23.3% with Grade 5, while 40% of the control group had a normal microbiota (Grade 1) (*p* < 0.0001). -Periodontal pathogens: The frequency of Tannerella forsythia (77%, *p* < 0.001) and Aggregatibacter actinomycetemcomitans (23%, *p* < 0.05) was significantly higher in the ESKD group.
Al-Nowaiser et al. (22)	Oral swabs, saliva and dental plaque.	-Culture-based methods: Mitis Salivarius agar, Rogosa agar, Sabouraud agar, etc. -Bacterial identification: Gram staining and biochemical tests were used, including carbohydrate fermentation and arginine and esculin hydrolysis.	-Taxa identified: Streptococcal species such as S. mitis, S. oralis, S. salivarius, S. vestibularis, S. sanguis, S. parasanguinis, S. gordonii, S. mutans, and S. sobrinus. Candida albicans and Lactobacilli were also evaluated. -Diversity indices: This study does not report “alpha” or “beta” diversity indices in the conventional manner, but rather presents the frequency of isolation and the proportion of each species relative to the total anaerobic count.	-Microbiology: Streptococcus mutans: The frequency of isolation and the total count were significantly lower in children with CKD (*p* = 0.002 and *p* = 0.03, respectively). Streptococcus parasanguinis: The frequency of isolation and its proportion were significantly higher in the CKD group (*p* = 0.03 and *p* = 0.02). Candida albicans: It was isolated in only 11 children in the control group (*p* = 0.0001), and was absent in children with CKD. Total aerobic count: It was significantly higher in children with CKD (*p* = 0.01).
Cesar Alves et al. (20)	Saliva samples (specifically unstimulated whole saliva) and oral biofilm (supragingival plaque).	Mass spectrometry using MALDI-TOF MS (Matrix-assisted laser desorption/ionization time-of-flight mass spectrometry) using the Biotyper 3.1 database.	-Reported taxa: The microorganisms with the highest incidence in saliva were Streptococcus salivarius (97%), Staphylococcus aureus (93%), Corynebacterium argentoctens (63%), Aggregatibacter actinomycetencomitans (60%), Acinetobacter ursingi (60%), Actinomyces dentalis (43%), and Tannerella forsythensis (43%). In oral biofilm, the most prevalent were Streptococcus salivarius (87%), Staphylococcus aureus (73%), Corynebacterium argentoctens (70%), Acinetobacter ursingi (50%), Aggregatibacter actinomycetencomitans (50%), and Pseudomonas pertucinogena (40%). Diversity indices: The sources provided do not mention alpha or beta diversity indices; instead, they report the frequency and percentage of incidence of specific species.	No differences were reported compared to a healthy control group, as the study focused exclusively on individuals diagnosed with CKD and periodontal disease (PD) to identify the microorganisms present in them.
Hoefer et al. (4)	Swabs from the back of the tongue.	16S rRNA gene sequencing (Next-generation sequencing).	-Taxa: Five main phyla were identified (Firmicutes, Proteobacteria, Bacteroidota, Actinobacteria, Fusobacteria), and the most abundant species were Streptococcus salivarius, Neisseria meningitidis, and Prevotella melaninogenica. -Diversity indices: Alpha diversity was assessed using the Shannon Index, and beta diversity was assessed using the weighted UniFrac method.	-Diversity: There were no significant differences in richness or alpha diversity (Shannon Index) between patients and their mothers (*p* > 0.05). Beta diversity showed that interindividual variation was greater than the differences between groups. -Composition (Phylum): The percentage of Proteobacteria was significantly higher in the CKD group (23.65% vs. 12.1%, FDR=0.037). -Relative abundance (Species): Neisseria meningitidis: Significantly increased in CKD patients (21.17% vs. 9.87%, FDR=0.0308). -Streptococcus parasanguinis: Significantly decreased in CKD patients (2.24% vs. 7.54%, FDR=0.0080). -Conclusion on the differences: The authors suggest that the differences in individual species could be attributed more to age or diet than to CKD itself.
Yang et al. (25)	Saliva samples (unstimulated) and dental plaque (supragingival)	The 16S rDNA high-throughput sequencing technique (PacBio platform).	-Taxa: The dominant phyla were Firmicutes and Proteobacteria; the primary genera identified were Streptococcus and Neisseria. -Diversity indices: The Chao1 and ACE indices (species richness) and the Shannon and Simpson indices (species diversity) were used.	-HSP with caries vs. HSP without caries: Patients with caries showed a greater abundance of Prevotella and Streptococcus mutans. They also exhibited a trend toward lower oral microbiota diversity before dental treatment. Treatment impact: Following systematic dental treatment, there was a significant increase in oral microbiota diversity (measured by the Shannon index, *p* < 0.05). Kidney-related clinical differences: The group with untreated caries showed a significant increase in urinary protein (PRO) at 3 months of follow-up, suggesting a higher risk of kidney involvement compared to the group that received dental treatment. The treated group showed a significant decrease in urinary white blood cells (UWBC).

The most frequently used microbiological method was 16S rRNA/rDNA gene sequencing (4, 21, 24, 25), followed by mass spectrometry (GC-MS or MALDI-TOF) (20, 23). Culture-based methods with biochemical tests and specific PCR assays were each used in only one study (22, 23).

#### 3.3.1 Microbial diversity and community structure

The studies evaluating alpha and beta diversity did not show a consistent direction of change across paediatric renal conditions. Pang et al. (21) reported significantly lower microbial richness and diversity in children with Henoch-Schönlein purpura nephritis (HSPN) than in thealthy controls. Specifically, the ACE and Chao richness indices were significantly lower in the disease group (*p* = 0.001 and *p* = 0.004, respectively), as were the Shannon and Simpson diversity indices (*p* = 0.001 and *p* = 0.005, respectively).

Conversely, Chen et al. (24) found significantly greater richness and diversity in children with Henoch-Schönlein purpura. The observed operational taxonomic unit richness was significantly higher in the disease group (*p* < 0.01), as was the Shannon diversity index (*p* < 0.0001). Differences in community structure were also identified by principal coordinates analysis and confirmed using ANOSIM (*p* = 0.0001).

In contrast, Hoefer et al. (4) observed no significant differences in alpha diversity between young patients with CKD and the comparison group (Shannon index, *p* > 0.05), while beta-diversity analysis indicated that interindividual variation was greater than the differences between groups.

Therefore, the available evidence did not demonstrate a uniform increase or decrease in oral microbial diversity in children with kidney disease.

#### 3.3.2 Differences in microbial taxa

Most studies identified differences in microbial composition between patients with renal disease and comparison groups; however, no single microbial signature was consistently reported across all studies. The most marked alteration was observed by Morozova et al. (23) in adolescents with end-stage kidney disease, of whom 46.7% presented Grade 4 dysbiosis and 23.3% presented Grade 5 dysbiosis. In comparison, 40% of the control group presented a normal microbiota, classified as Grade 1, and the overall distribution of dysbiosis grades differed significantly between groups (*p* < 0.0001). Tannerella forsythia was detected in 77% of patients with end-stage kidney disease (*p* < 0.001), whereas Aggregatibacter actinomycetemcomitans was detected in 23% (*p* < 0.05).

The results for individual taxa were frequently contradictory. For example, Al-Nowaiser et al. (22) reported a higher frequency and proportion of Streptococcus parasanguinis in children with CKD (*p* = 0.03 and *p* = 0.02, respectively). Whereas Hoefer et al. (4) found a significantly lower relative abundance of this species in patients with CKD than in the comparison group (2.24% versus 7.54%; FDR-adjusted *p* = 0.0080). Hoefer et al. suggested that differences in individual species might be more strongly influenced by age or diet than by CKD itself.

Similarly, Pang et al. (21) reported lower abundance of Prevotella and Campylobacterales and a higher abundance of Eikenella in children with Henoch-Schönlein purpura nephritis (*p* < 0.05). In contrast, Chen et al. (24) observed enrichment of *Prevotella*, *Neisseria* and *Veillonella* in children with Henoch-Schönlein purpura (22, 23).

Contradictory findings were algo oberseved for *Candida albicans*. Morozova et al. (23), identified Candida among the opportunistic microorganisms detected in adolescents with end-stage kidney disease. Conversely, Al-Nowaiser (22) reported that *Candida albicans* was absent in children with CKD and was isolated only in the control group (*p* = 0.0001).

## 4 Discussion

The main finding of this systematic review was that children and adolescents with kidney disease may present alterations in the oral microbiome, although the available studies did not identify a consistent microbial signature. The direction of the changes in microbial diversity and in the relative abundance of individual taxa differed across studies. The most marked dysbiosis was reported in adolescents with end-stage kidney disease, whereas the study examining the tongue microbiome of young patients with CKD found no significant differences in alpha diversity. In addition, opposing findings were reported for taxa such as *Streptococcus parasanguinis*, *Prevotella* and *Candida*. Overall, the current evidence supports the possible presence of oral ecological alterations in paediatric kidney disease, but not a reproducible disease-specific microbial profile.

The clinical and methodological heterogeneity of the included studies may partly explain these inconsistencies. The study populations differed in renal diagnosis, disease stage, dialysis or transplantation status, age, dietary restrictions and pharmacological exposure. Furthermore, saliva, tongue coating, oral swabs, dental plaque and gingival crevicular fluid represent distinct ecological niches and may therefore show different microbial profiles. The microbiological methods also varied considerably, including sequencing, culture-based techniques, PCR and mass spectrometry. These differences limited direct comparisons between studies and precluded a quantitative synthesis.

Age and diet may be particularly relevant when interpreting changes in individual taxa. Hoefer et al. (4) suggested that the differences observed in species such as *Streptococcus parasanguinis* might be more strongly influenced by age or dietary factors than by CKD itself. Consequently, microbial alterations identified in a specific renal phenotype or oral niche should not be directly extrapolated to all children and adolescents with kidney disease. These potential confounding factors should be controlled more consistently in future studies.

In children and adolescents with chronic kidney disease (CKD) and/or on peritoneal dialysis, saliva is a key component of the oral-systemic axis, as mentioned in the introduction, because it also regulates pH, substrate clearance and oral biofilm stability (9, 26). Consequently, changes in salivary quantity or composition may provide a biologically plausible mechanism linking renal dysfunction with alterations in the oral microbial ecosystem.

CKD may be associated with salivary metabolomic changes, hyposalivation and alterations in salivary composition (9, 27, 28). There may also be an increase in salivary urea, whose metabolism by urease-positive bacteria generates ammonium and thus promotes relative pH alkalisation, with the potential to modify the local microbial ecology (9, 29, 30), favour more persistent biofilms and a transition towards oral dysbiosis (26, 27) as described in our results and so well defined by Sergeevna Morozova et al. (23).

This mechanism is consistent with the findings of Al Nowaiser et al. (22), who reported higher salivary urea levels and buffering capacity in children with CKD, together with a lower concentration of *S. mutans* than in healthy controls. Nevertheless, the observational design of the study does not allow a causal relationship between these biochemical alterations and microbial composition to be established.

This oral dysbiosis in children and adolescents with CKD may be related to a higher inflammatory burden (4, 5, 9). From a clinical perspective, the combined assessment of salivary biomarkers, such as urea and pH, and microbiological indicators could potentially contribute to the identification of oral ecological alterations in this population. However, their usefulness as non-invasive biomarkers should be confirmed in longitudinal studies (29, 31, 32).

On the other hand, comparison between different phenotypes of kidney disease suggests that the composition of the oral microbiome is not uniform throughout the clinical course of the disease. In adults, differences in beta-diversity have been observed between early CKD, advanced CKD, and end-stage renal disease on replacement therapy, with a tendency towards enrichment of periodontopathogenic taxa in advanced stages and differentiated community profiles in haemodialysis (29, 30). Consistently, salivary analyses in CKD have described distinct microbial signatures compared to healthy controls, with variations in genera potentially linked to inflammation and uremic metabolism (32). Although these adult findings provide a plausible framework for interpreting the paediatric results, they should not be directly extrapolated to children because age, renal aetiology, treatment exposure and microbiome maturation differ substantially between populations.

Of all the methods used to analyse the oral microbiome, 16S rRNA/rDNA gene sequencing is the most widely used, according to our results, which coincide with those of the systematic review by D'Agostino et al. (33). However, differences in the amplified regions, sequencing platforms, bioinformatic pipelines and taxonomic databases may influence the microorganisms detected and contribute to the variability observed between studies.

In haemodialysis, an association has also been observed between oral microbial structure and treatment exposure time, supporting a cumulative effect of the uraemic environment and dialysis therapy on oral ecology (30). In paediatrics, the findings are more nuanced and seem to depend on the oral niche analysed. Studies of the lingual microbiome in children and adolescents with CKD show relative ecological stability compared to healthy controls and a limited response to intensive prophylactic interventions, suggesting resilience of this niche at an early age (4, 5). However, this apparent stability does not exclude alterations in saliva or dental plaque, especially in contexts with salivary biochemical changes such as peritoneal dialysis (28). Overall, the evidence supports the existence of differential compositional patterns between types of kidney disease (non-dialysis CKD, haemodialysis, peritoneal dialysis, and transplantation), although methodological heterogeneity persists, limiting comparison.

Similarly, a longitudinal clinical trial demonstrated that an intensive oral prophylaxis programme did not produce clinically relevant changes in the composition of the tongue microbiome, confirming its ecological stability in paediatric patients with CKD (4).

On the other hand, caries is interpreted as a dysbiosis of the oral ecosystem rather than the isolated presence of a single pathogen (33). Although this review did not aim to analyse the influence of the oral microbiome on the development of caries, some authors such as Yang et al. (25) reported changes in urinary parameters following dental treathment, whereas Al-Nowaiser et al. (22) found a higher proportion of caries-free children in the CKD group than among healthy controls. These findings should be interpreted cautiously because the studies had different objectives and designs, and they do not demonstrate that dental treatment or modification of the oral microbiome improves renal function.

This systematic review has several limitations. The number of included studies was small, and many had limited sample sizes. Furthermore, the populations were clinically heterogeneous, including different renal diagnoses, CKD stages, dialysis modalities and transplantation status. Relevant confounding factors, such as age, diet, oral hygiene, caries, medication and immunosuppressive therapy, were not consistently reported or controlled. The methodological quality was also variable, with most studies presenting a moderate risk of bias and two studies classified as having a high risk of bias.

The statistical analysis of the available evidence was also limited. The included studies used different diversity indices, taxonomic outcomes and statistical tests, and several reported *p*-values without effect estimates or confidence intervals. Together with the heterogeneity in oral sampling sites and microbiological methods, these differences prevented a meta-analysis and limited the assessment of the magnitude and precision of the associations. Consequently, the findings should be interpreted cautiously, as the current evidence is insufficient to establish a reproducible microbial signature or causal relationship in paediatric kidney disease.

## 5 Conclusions

There is an urgent need to establish comprehensive protocols for oral healthcare and protection in paediatric nephrology units in hospitals. More homogeneous studies with larger sample sizes are needed to obtain conclusive data that will allow us to determine, through parameters such as urea, pH or oral microbiota agents, their relationship with the different stages of CKD and its severity.

## Data Availability

The original contributions presented in the study are included in the article/Supplementary Material, further inquiries can be directed to the corresponding author.
